# Late-Onset Musical Obsession: A Case Report of an Elderly Patient Treated in an Outpatient Psychiatry Clinic

**DOI:** 10.7759/cureus.71369

**Published:** 2024-10-13

**Authors:** Yuyuan Tan, Aaron Meng, Kiranjeet Kaur

**Affiliations:** 1 Psychiatry, Ng Teng Fong General Hospital, Singapore, SGP

**Keywords:** earworm, late-onset, musical obsession, obsessive-compulsive disorder (ocd), stuck song syndrome

## Abstract

Musical obsessions are a form of involuntary musical imagery that may occur in the context of obsessive-compulsive disorder and patients presenting with this symptom are usually in their early adulthood. This case highlights the unusual presentation of musical obsessions in an elderly patient and the subsequent evaluation and treatment given. It is important to recognize and distinguish this from other symptoms such as auditory hallucinations with musical content and palinacousis as this will affect the subsequent clinical assessment and management required.

## Introduction

Musical obsessions, also termed “stuck song syndrome”, can cause significant anxiety and dysfunction similar to that seen in other forms of obsessive-compulsive disorder (OCD) [1]. They are characterized by the presence of repetitive, intrusive and involuntary musical imageries causing distress and functional impairment [2].

The content of these musical obsessions can vary, generally manifesting as fragments of sound or music heard in the past and may run an episodic or continuous course [3]. These repetitive tunes or song fragments occur without conscious or deliberate efforts to experience them [4]. Attempts to block these out are usually unsuccessful and result in heightened anxiety. These features are in keeping with the definition of obsessions under the Diagnostic and Statistical Manual of Mental Disorders (DSM-5) criteria for OCD [5].

It is important to differentiate musical obsessions from other forms of sound-related disturbances such as “earworms”, musical hallucinations and palinacousis, as this will have implications on the diagnosis and subsequent treatment.

“Earworms”, also known as sticky tunes, are a fairly common phenomenon amongst the general population and do not reach the level of an obsession [2]. These are usually self-resolving, triggered by a recent exposure to a piece of music and described by majority as a pleasant or neutral experience [6].

Musical hallucinations are a form of auditory hallucinations, where musical or song fragments are experienced as originating from external to the head, in the absence of any actual stimuli and are beyond voluntary control. A study found that compared to involuntary musical imagery, subjects with musical hallucinations described their experiences as having less lyrical content and are usually unfamiliar in nature [7]. These are more commonly seen in the older age group with a female preponderance, and possible aetiologies range from neurological, psychiatric, and substance-related to hearing impairment [8]. 

Another differential is palinacousis. This is an uncommon symptom usually associated with temporal lobe dysfunction and epilepsy [9]. It is a form of auditory illusion, occurring as a sound fragment perseverating after a recent auditory stimulus has ended [10]. Palinacousis is episodic and not usually associated with other psychiatric or mood symptoms [9].

Cases of musical obsessions are reported to be uncommon [11]. In a cross-sectional study, musical obsessions were reported in about 5% of individuals with OCD [12]. Majority of the cases of musical obsessions reported thus far had also been more frequently observed in patients in the adolescent age group or in their 30s [2,13]. This case report study seeks to share an uncommon presentation of musical obsession with late-life onset in a patient with no pre-existing psychiatric illness or underlying organic cause identified. 

## Case presentation

Ms. A was a 64-year-old lady at the time of presentation. She had initially presented to her primary care physician with symptoms of insomnia of about two months' duration, with difficulties initiating and maintaining sleep. She was also noted to be anxious over her sleep difficulties. Ms. A was then prescribed oral amitriptyline 10mg once at night and oral zopiclone 7.5mg once at night to use as necessary for her insomnia complaints.

However, her sleep and anxiety symptoms did not improve. Instead, she reported increasing anxiety over her sleep difficulties and started hearing repeated chanting songs. Ms. A was then referred to and seen in the Psychiatry specialist clinic a month later. Her symptoms had persisted and did not improve after discontinuation of her initial medications from her primary care physician. 

On further assessment in the Psychiatry clinic, Ms. A described experiencing recurrent and intrusive religious chanting being repeated inside her head. At times, she also experienced songs she had heard on the radio, without religious theme, replaying in her head repeatedly. These religious chanting and songs were familiar tunes she had heard before and were in her native language of Mandarin.

She reported that these intrusive songs were heard on a daily basis and persistent throughout the day and night. Although there was no clear external auditory component, she experienced these song fragments as being very vivid and distressing, further affecting her sleep at night and leading up to two separate emergency department consults in a state of anxiety when she was unable to block them out. During her emergency department consults, she was prescribed oral lorazepam 0.5mg once a day, not exceeding two days for rapid relief of her anxiety.

Her ability to carry out her daily chores and caregiving duty for her grandchild was also affected as a result of the intense anxiety and frustration when she was unable to block out the recurrent intrusive songs. She did not have any other obsessions or compulsions. There was no pervasive low mood or anhedonia, and she was not suicidal. There was no other hallucinatory experience in other sensory modalities, no delusions and no history of manic symptoms. She did not experience any cognitive decline.

Ms. A's other medical history included hypertension, for which she had been taking oral spironolactone, oral telmisartan and oral beta-nicardia long-standing prior to the onset of her symptoms. She did not have any other chronic medical illness nor prior history of psychiatric illness. There was no history of fits or other neurological symptoms. She did not consume any traditional medications. She also did not have a history of alcohol intake, smoking or illicit drug use. There was no family history of psychiatric illness such as obsessive-compulsive disorder.

Ms. A was married and living with her husband. She did not work and had been a housewife over the years. Her daily routine included looking after her grandchild at home and taking care of the household chores. She shared a good relationship with her family and there was no external stressor prior to the onset of her insomnia complaints preceding the musical obsession symptoms. In terms of premorbid personality, Ms. A was described to be slightly anxious but had been able to cope independently through various life events and changes over the years. She was otherwise sociable and outgoing. She did not have any particular religion nor strong preference in music. 

On mental state examination at initial presentation, Ms. A was observed to be tense and anxious, ruminating over her symptoms and repeatedly seeking reassurance over her condition. Her speech was slightly rapid and verbose. There was no observed speech or thought disorder otherwise. There were no other hallucinatory experiences and no delusions. Musical obsessions were reported to be present but did not affect her ability to converse. She was not suicidal. No focal neurological deficit was noted during the physical examination.

Laboratory investigations were done to rule out other organic causes for her presentation. Her full blood count, renal function test, liver panel, thyroid panel, calcium panel, vitamin B12 and vitamin D levels were normal. Syphilis and Human Immunodeficiency Virus (HIV) screen were not done as she was unlikely to have prior exposure based on her social history. 

Ms. A was further reviewed by a Neurologist and Otolaryngologist (ENT) to exclude other possible neurological or hearing-related causes. Magnetic Resonance Imaging (MRI) of the brain showed the presence of age-appropriate cerebral involution as well as bilateral periventricular and deep white matter chronic microvascular ischemic changes. Electroencephalogram (EEG) done was normal. Her hearing assessment did not identify any significant hearing impairment.

Ms. A was started on oral sertraline, which was titrated according to her symptom response up to 150mg once a day. She reported a reduction of the duration and intensity of musical obsessions and associated anxiety levels but was unable to achieve complete resolution. With higher doses of sertraline beyond 150mg once a day, she reported no further improvement and instead experienced some changes in the nature of the musical obsessions to have a more “echo-like” quality and hence capped at that dosage. She was also prescribed quetiapine up to 150mg once a day for insomnia and augmentation of control of her musical obsession and associated anxiety symptoms. Psychological therapy was discussed but Ms. A was not keen due to personal preference. 

Ms. A reported subjectively that the duration and intensity of her musical obsessions had lessened significantly by up to 90% with treatment and follow-up over the next one year and had been maintained on the same dose of medications subsequently. She no longer experienced any musical obsessions for most part of her day.

There were still residual mild musical obsessions of the similar religious chanting songs occasionally when the external surroundings were quiet when she was alone or at night. The residual musical obsessions would be reduced when she was engaged talking to others, playing mobile phone games or when she was actively listening to the radio or watching television. Her anxiety and insomnia symptoms had also resolved. She was no longer in distress and was able to resume her daily activities and lifestyle. 

## Discussion

Ms. A’s presentation with recurrent intrusive sound fragments experienced as occurring inside the head and beyond voluntary control was more in keeping with that of a musical obsession than hallucination. Moreover, the severity and persistence of the musical obsessions triggering significant anxiety and disrupting her daily routine indicated that this was not just an “earworm”, as such requiring further clinical attention.

Currently, there is no clear consensus on the definition of age of onset for late-onset musical obsessions. Age cut-offs referenced in other studies of late-onset OCD ranged from 40 to 60 years old [14-16]. The nature of obsessions more commonly reported in a review of late-onset OCD were those relating to dirt and contamination, the need to know, and sexual images [17]. In view of the uncommon presentation of this symptom in an elderly patient with no pre-existing medical or psychiatric illness, further medical evaluation was necessary to exclude other possible underlying organic causes.

Whilst the exact cause of musical obsessions is still unclear, it is postulated to be similar to other forms of OCD. Changes on EEG suggestive of abnormal functioning of the fronto-basal regions and fronto-temporal circuits of the brain were reported in one case study on musical obsession [18]. In a systematic review of late-onset OCD, abnormal brain findings were found in 66.66% of cases, most commonly affecting the frontal regions and basal ganglia [17]. 

In another case report of a 51-year-old patient with musical obsessions, the patient was noted to have underlying hearing impairment from otosclerosis [19]. In Ms. A’s case, her ear and hearing evaluation did not reveal any significant disorder.

The treatment of musical obsessions is similar to that for OCD [2]. Common psychotropic medications recommended include the use of selective serotonin reuptake inhibitors and clomipramine. Cognitive behavioral therapy is also recommended [2]. Other non-pharmacological interventions such as engaging in cognitively challenging tasks such as puzzles may also alleviate the symptoms [20].

In Ms. A’s case, she responded to treatment with sertraline and quetiapine augmentation. She was not keen for psychological therapy but as she resumed her social activities with better symptom control after treatment, she reported further reduction of her symptoms when she was actively engaged in conversations or cognitive tasks such as playing mobile phone games.

Reports of musical obsessions among the geriatric population are uncommon. This condition may also be under-recognized due to limitations in current available screening and diagnostic tools. As such, this may be an area for further research and development in future studies with more similar cases being identified. 

## Conclusions

Ms. A's case study adds to the limited knowledge of this rare presentation. Musical obsessions can be distressing and cause functional impairment without treatment, and it is important to differentiate it from other similar musical experiences to ensure appropriate treatment is given.
